# Cellular and Matrix Response of the Mandibular Condylar Cartilage to Botulinum Toxin

**DOI:** 10.1371/journal.pone.0164599

**Published:** 2016-10-10

**Authors:** Eliane H. Dutra, Mara H. O’ Brien, Alexandro Lima, Zana Kalajzic, Aditya Tadinada, Ravindra Nanda, Sumit Yadav

**Affiliations:** 1 Department of Orthodontics, University of Connecticut Health Center, Farmington, Connecticut, United States of America; 2 Department of Oral and Maxillofacial Diagnostic Sciences, University of Connecticut Health Center, Farmington, Connecticut, United States of America; 3 Department of Oral and Maxillofacial Radiology, University of Connecticut Health Center, Farmington, Connecticut, United States of America; University of Alabama at Birmingham, UNITED STATES

## Abstract

**Objectives:**

To evaluate the cellular and matrix effects of botulinum toxin type A (Botox) on mandibular condylar cartilage (MCC) and subchondral bone.

**Materials and Methods:**

Botox (0.3 unit) was injected into the right masseter of 5-week-old transgenic mice (Col10a1-RFPcherry) at day 1. Left side masseter was used as intra-animal control. The following bone labels were intraperitoneally injected: calcein at day 7, alizarin red at day 14 and calcein at day 21. In addition, EdU was injected 48 and 24 hours before sacrifice. Mice were sacrificed 30 days after Botox injection. Experimental and control side mandibles were dissected and examined by x-ray imaging and micro-CT. Subsequently, MCC along with the subchondral bone was sectioned and stained with tartrate resistant acid phosphatase (TRAP), EdU, TUNEL, alkaline phosphatase, toluidine blue and safranin O. In addition, we performed immunohistochemistry for pSMAD and VEGF.

**Results:**

Bone volume fraction, tissue density and trabecular thickness were significantly decreased on the right side of the subchondral bone and mineralized cartilage (Botox was injected) when compared to the left side. There was no significant difference in the mandibular length and condylar head length; however, the condylar width was significantly decreased after Botox injection. Our histology showed decreased numbers of Col10a1 expressing cells, decreased cell proliferation and increased cell apoptosis in the subchondral bone and mandibular condylar cartilage, decreased TRAP activity and mineralization of Botox injected side cartilage and subchondral bone. Furthermore, we observed reduced proteoglycan and glycosaminoglycan distribution and decreased expression of pSMAD 1/5/8 and VEGF in the MCC of the Botox injected side in comparison to control side.

**Conclusion:**

Injection of Botox in masseter muscle leads to decreased mineralization and matrix deposition, reduced chondrocyte proliferation and differentiation and increased cell apoptosis in the MCC and subchondral bone.

## Introduction

Temporomandibular joint disease (TMD) is a diverse group of conditions involving the temporomandibular joint (TMJ) and its adjacent tissues. The etiology behind this disorder is complex, but the symptoms are similar and are commonly manifested as pain in the orofacial region; which impacts significantly the patient’s quality of life [1,2]. Treatments for TMD include conservative therapies such as relaxing the masticatory muscles by limiting jaw movements, parafunctional habit modification, oral splints, soft diet, moist heat and/or ice therapy, physical therapy and medications [3]. However, it is believed that about 30% of patients do not respond well to non-invasive treatments.

Recently, injection of Botulinum Neurotoxin Type A (Botox) into the masticatory muscles have been used to treat myofascial pain syndrome (including bruxism), temporomandibular joint disorders (TMDs), tension headache and chronic migraine headache [4,5]. In migraine type-headaches, bruxism and TMDs, masticatory muscles innervated by trigeminal nerves are targeted. Botulinum toxin works by blocking the cholinergic transmission and acetylcholine release at the neuromuscular junction, resulting in temporary flaccid paralysis of the injected muscle [6]. Furthermore, Botox is being used as a cosmetic procedure to reduce the thickness and tonicity of the masseter muscle and create a slimmer oval shape face [7,8].

However, Botox injections into the masticatory muscles could potentially unload the mandible and cause anatomical changes as well as osteopenia of the mandibular ramus, alveolar bone and subchondral bone. In fact, clinical and animal studies have consistently confirmed the deleterious effect of Botox injections into the masseter in the mandibular ramus and condyle [9–12], but there are no investigations with the aim of understanding the cellular mechanisms behind the osteopenia caused by this type of treatment. Furthermore, to our knowledge, there are no studies evaluating the cellular effects of Botox injection into the masticatory muscles on the mandibular condylar cartilage (MCC) and subchondral bone using transgenic reporter mice. The TMJ has the capacity to adapt to external stimuli and loading changes can affect the position of condyles as well as the structural and cellular components of the MCC. It has been shown that altering masticatory loading can cause damaging effects in the MCC, such as decreased cartilage thickness, chondrocyte proliferation and protein expression [13–15].

The objectives of this study were 1) to quantify the cellular changes in the MCC with injection of Botox in the masseter muscle; 2) tissue level changes in the MCC and subchondral bone.

## Materials and Methods

### Mice

All animal experimental procedures were approved by the Institutional Animal Care Committee of the University of Connecticut Health Center (UCHC). All the animal experiments followed the ARRIVE guidelines [16]. We used 5-week-old females (n = 9) and males (n = 4) transgenic mice (Col10a1) on a CD-1 background. The transgene (Col10a1-RFPcherry) used in this study have been previously described [17]. Col10a1-RFPcherry are expressed in the hypertrophic zone of the mandibular condylar cartilage, so we used this model in order to study temporal changes in Col10a1 expression after Botox injection.

### Experimental Procedure

Mice were anesthetized with ketamine (90 mg/kg) and Xylazine (13 mg/kg) before the injections. Botox (Botox^®^; OnabotulinumtoxinA; Allergan, Plc; Parsippany-Troy Hills, NJ, USA) was injected into the right side masseter of transgenic mice (0.3 unit, volume of 30 μl). This dose was injected in a single injection point in the inferior portion of the superficial masseter muscle. The left side masseter did not receive any injection and served as intra-animal control. We performed unilateral injection to have an internal control and because this approach would provide a more uniform response by the experimental mice. We injected 0.9% saline solution (30 μl) into the right masseter of four animals in our first pilot experiment, but since we did not observe differences in the histological features between saline injected and control side (S1 File and S1 Fig), we decided to exclude this group from our experiments due to the scarce number of transgenic mice.

All the animals were intraperitoneally injected with calcein (10 μg/kg body weight) and alizarin complexone (10 μg/kg body weight) alternatingly at day 7, day 14 and day 21. In addition, mice were injected with EdU (5-ethnyl-2’-deoxyuridine, Life Technologies, Grand Island, NY, USA), in a concentration of 30mg/kg body weight, 48 and 24 hours before euthanasia. Animals were weighed weekly and animals gained about 20% of its weight at the end of experiments. Mice were euthanized 30 days after Botox injection by CO_2_ asphyxiation.

### Tissue Preparation and Histology

The right and the left mandibles were dissected free by cutting the muscular attachment without scrapping the cartilage of the condyle. Mandibles were fixed in 10% Formalin for 24 hours. Fixed undecalcified mandibles were placed in 30% sucrose overnight and embedded in frozen specimen embedding medium (Shandon Cryomatrix^™^, Thermo Scientific, Pittsburgh, PA, USA). Frozen sagittal sections of the condyles (5 μm) were performed using the Kawamoto method [18].

### Micro-CT

Mineralized cartilage and subchondral bone in the control and Botox were analyzed using micro-computerized tomography (SCANCO Medical AG, Brüttisellen, Switzerland). The samples were scanned in 70% ethanol, one at a time, with high resolution in a 16mm holder. Serial tomographic projections were acquired at 55kV and 145μA, with a voxel size of 6μm and 1000 projections per rotation collected at 300000μs. The DICOM images were transferred, segmented and reconstructed using the mimics software (Materialise, Belgium). In order to distinguish calcified tissue from non-calcified tissue, an automated algorithm using local threshold segmented the reconstructed grey scale images. Bone mineral density (BMD (mg/cc)), bone volume fraction (BVF (%)), trabecular thickness (Tb.Th (μm)), and trabecular spacing (Tb.Sp (μm)) were determined.

### Morphometric Measurement

Radiographs of mandibles were taken with a MX20 Radiography System (Faxitron X-Ray LLC, Lincolnshire, IL, USA) at a 26Kv for 5 seconds. Morphometric measurements in control and Botox injected side mandibles were accomplished using Digimizer^®^ Image software (MedCalc Software, Mariakerke, Belgium). The following measurements were performed: mandibular length (condylion to incisor process); condyle head length (perpendicular distance from condylion to a line traced from the sigmoid notch to the deepest point in the concavity of the mandibular ramus) and; condyle head width (distance from the most anterior to the most posterior point of the condylar articular surface). Measurements were done in triplicate and the average for each side of respectively mice was calculated.

### Histological Staining

The first step was to image for Col10a1-RFPcherry and bone labels alizarin complexone (red) and calcein (green). Baseline imaging of the sections was performed with the observer ZI fluorescent microscope (Carl Zeiss, Thornwood, NY, USA) using a RFPcherry filter that was also used for detecting alizarin complexone staining (mCherry, Chroma Cat 49009ET, EX: 560/40, EM: 630/75). In the next step, the coverslip was removed by soaking in PBS and the sections were stained for EdU (ClickiT^®^ EdU Alexa Fluor 555 HCS kit, Life Technologies, Grand Island, NY, USA) and imaged. Subsequently, sections were stained for Tartrate Resistant Acid Phosphatase (TRAP) using ELF97 (Life Technologies, Grand Island, NY, USA), which generates a yellow fluorescent signal. After imaging for TRAP, the coverslip was removed and the same slide was stained for Alkaline Phosphatase (AP) activity using a fluorescent fast red substrate (Sigma-Aldrich, St. Louis, USA) and DAPI (Thermo Fisher Scientific, Waltham, MA, USA) and re-imaged. Finally the slide was rinsed in distilled water, stained with toluidine blue, and reimaged.

Different slides were also imaged for Safranin O (IHC WORLD, LLC; Ellicott City, MD, USA) following the manufacturer’s protocol. In addition, immunohistochemistry for pSMAD1/5/8 (EMD Millipore, Billerica, MA, USA) and VEGF (ABCAM, Cambridge, MA, USA) were performed. Cell apoptosis was evaluated by TUNEL staining (DeadEnd^™^ Fluorometric TUNEL System, Promega, Madison, WI, USA).

Histological analysis and quantification were described in S2 File and S2 Fig.

### Statistical analysis

Descriptive statistics were used to examine the distribution of bone volume fraction, trabecular thickness, trabecular spacing, morphometric measurements (mandibular length, condylar length, condylar width) and histological analyses. Outcomes were compared between the Botox injected side and the control side. Statistically significant differences among means were determined by paired t-test. All statistical tests were two sided and a p-value of < 0.05 was deemed to be statistically significant. Statistical analyses were computed using GraphPad Prism (GraphPad Software, Inc, La Jolla, CA, USA).

## Results

### Botox injection leads to decrease in tissue density and bone volume

**Fig 1:**
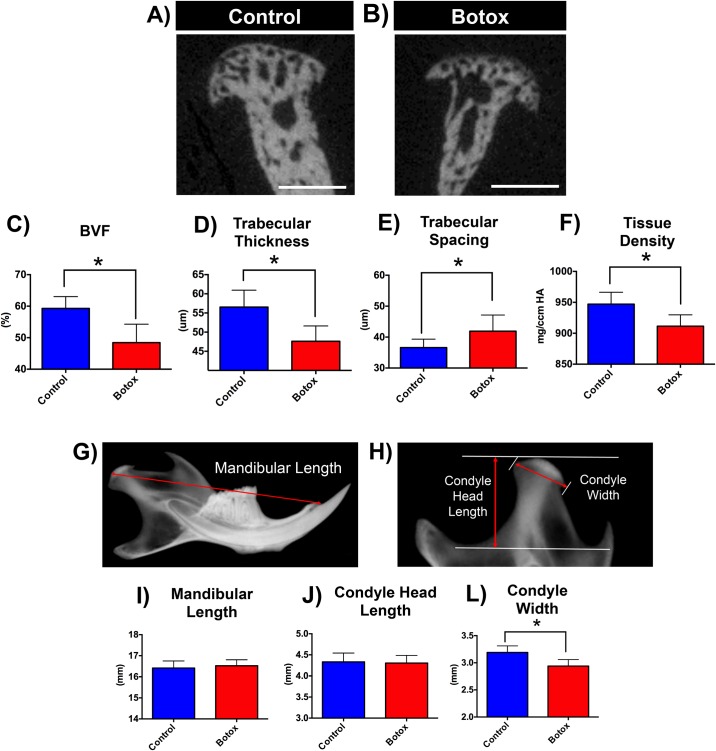
Reduced bone volume and density and reduced condyle width at the Botox injected side. **Coronal** micro-CT images of condyles of control **(A)** and Botox **(B)** injected side masseter 4 weeks after unilateral Botox injection. Quantification of bone parameters: **C)** BVF—bone volume fraction, **D**) Trabecular Thickness, **E)** Trabecular Spacing, **F)** Tissue Density. Morphometric measurements **(G-H)** performed in Faxitron xray images of control and Botox injected side mandibles: **I)** Mandibular Lengh, **J)** Condyle Head Length, **L)** Condyle Width. Histograms **(C-F, I-L)** represent means ± SD for n = 13 *per group*. *Significant difference between control and Botox injected side (p < 0.05). Scale bar = 500μm.

We used Micro-CT analysis to compare bone volume and quality of the mineralized cartilage and subchondral bone between Botox injected side condyle and control (Fig 1A and 1B). There was a significant decrease in bone volume in the Botox injected side in comparison to control, as revealed by a 21.44% decrease in BFV (p < 0.05, Fig 1C), as well as a 17.4% decrease in Trab. Thickness (p < 0.05), Fig 1D) and an 18.37% increase in Trab. Spacing (p < 0.05, Fig 1E). Furthermore, we observed a significant decrease in tissue density (4.10% decrease, p < 0.05, Fig 1F) at the injected side after unilateral Botox injection.

### Botox injection leads to decrease in condylar width

We performed morphometric measurements of Faxitron xray images of Botox injected side mandibles and controls (Fig 1G and 1H) and found no difference in mandibular length (Botox: 16.50 ± 0.32mm vs Control: 16.55 ± 0.29mm, p > 0.05, Fig 1I) or condyle head length (Botox: 4.25 ± 0.14mm vs Control: 4.38 ± 0.22mm, p > 0.05, Fig 1J) between Botox and control sides. However, there was a significant decrease in condyle width in Botox injected side (Botox: 2.95 ± 0.13mm vs Control: 3.19 ± 0.14mm, p < 0.05, Fig 1L) when compared to control.

### Botox leads to decrease in bone turnover

**Fig 2:**
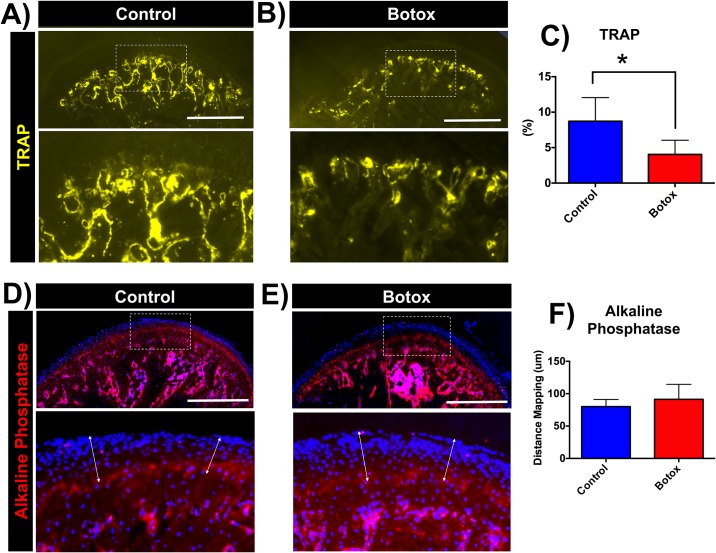
Decrease in osteoclastic activity and mineralization at the Botox injected side condyle. Sagittal sections of control (left side) **(A)** and Botox (right side) **(B)** injected side condyles stained for TRAP. **C)** Quantification of TRAP positive pixels (yellow) over subchondral bone area. Sagittal sections stained for alkaline phosphatase, control **(D)** and Botox injected side **(E)**. **F)** Quantification of distance mapping. Histograms **(C,F)** represents means ± SD for n = 7 *per group*. * Significant difference between control and Botox injected side (p < 0.05). No significant difference between control and Botox injected side for alkaline phosphatase distance mapping **(F)**. Scale bar = 500μm.

**Fig 3:**
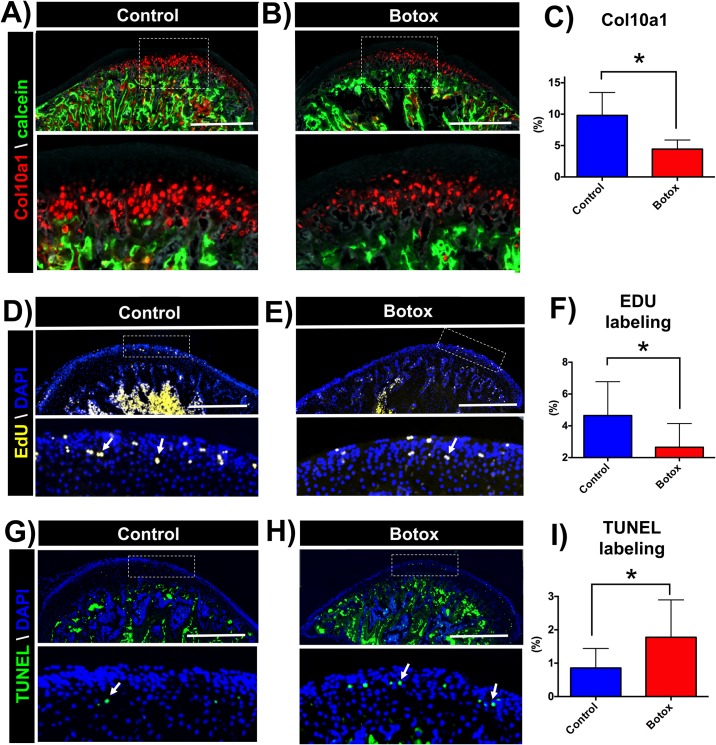
Reduced Col10a1 positive cells, decreased cell proliferation and increased cell apoptosis at the MCC of Botox injected side. Representative sagittal sections of condyles of transgenic mice (Col10a1-RFPcherry), control **(A)** and Botox **(B)** injected side. **C)** Quantification of Col10a1 positive pixels (red) over MCC area. Sagittal sections of control **(D)** and Botox **(E)** injected side condyles stained for EdU. **F)** Quantification of EdU positive pixels (yellow) over DAPI positive pixels (blue) at the proliferative zone. TUNEL staining in sections of control **(G)** and Botox **(H)** injected side condyles. Histograms **(C-I)** represents means ± SD for n = 7 **(C-F)** and n = 5 **(I)** *per group*. * Significant difference between control and Botox injected side (p < 0.05). Scale bar = 500μm.

Then we aimed to understand the mechanisms correlated with bone loss by evaluating bone remodeling. We studied osteoclast activity in the subchondral bone of Botox injected and control side condyles by TRAP staining (Fig 2A and 2B). Our quantification showed significant lower percentage of TRAP positive pixels (p < 0.05) in the injected side in comparison to control (Fig 2C), suggesting a low bone turnover. We next studied mineralization and we detected the alkaline phosphatase staining layer at the tidemark (mineralization zone) of the MCC in both sides analyzed (Fig 2D and 2E). The alkaline phosphatase distance mapping between Botox injected and control side was not statistically different (Botox: 87.52 ± 23.46 μm vs Control: 81.25 ± 10.04 μm, p > 0.05, Fig 2F). However, calcein labeling suggested reduced mineral deposition in the subchondral bone of Botox injected side condyle (Fig 3A and 3B).

### Botox injection leads to decrease in chondrocyte proliferation and differentiation and increases cell apoptosis

Our histology showed Col10a1 positive cells in the hypertrophic zone of the MCC, in both control and Botox injected side condyles (Fig 3A and 3B). However, quantification of Col10a1 positive pixels suggested a significant decrease in the number of Col10a1 cells in the Botox injected side (51.40% decrease, p < 0.05, Fig 3C). In addition, we observed EdU positive cells in the proliferative and prehypertrophic of the MCC in both side condyles (Fig 3D and 3E). Quantification of EdU positive pixels at the proliferative zoned revealed a significant lower percentage of EdU positive pixels in the injected side in comparison to control (Botox: 2.17 ± 1.44%, Control: 3.86 ± 2.16, p < 0.05, Fig 3F), suggesting decreased cell proliferation after unilateral Botox injection. In both groups, TUNEL positive cells were observed in the subchondral bone and proliferative layer and prehypertrophic layer of MCC (Fig 3G and 3H). However, in the Botox injected side, both in the subchondral bone and MCC, there were significantly more (p < 0.05) positive TUNEL stained cells when compared to the control (Fig 3I).

### Botox leads to decrease in proteoglycan secretion

**Fig 4:**
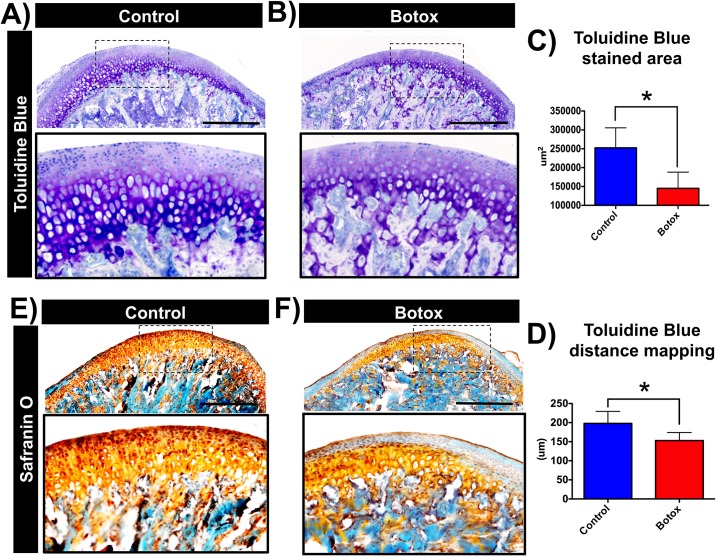
Reduced proteoglycan secretion at the Botox injected side MCC. Sagittal sections of control **(A)** and Botox **(B)** injected side condyles stained for toluidine blue. **C-F)** Quantification of toluidine blue stained area and distance mapping at the MCC. Safranin O staining for control **(E)** and Botox injected side condyles **(F)**. Histograms **(C-D)** represent means ± SD for n = 7 *per group*. * Significant difference between control and Botox injected side (p < 0.05). Scale bar = 500μm.

In addition, we analyzed the non-mineralized region of the MCC by Toluidine blue (TB) and Safranin O staining. TB staining revealed reduced proteoglycan secretion at the Botox injected side (Fig 4A and 4B) as illustrated by significant decreased TB stained area (Botox: 236309 ± 151651 μm^2^ vs Control: 242782 ± 49585 μm^2^, p < 0.05, Fig 4C) and shorter TB distance mapping (Botox: 161.1 ± 19.89 μm vs Control: 191.7 ± 29.05 μm, p < 0.05, Fig 4D). Furthermore, Safranin O staining showed a marked decrease of glycosaminoglycans in the MCC of the Botox injected side when compared to control side (Fig 4E and 4F)

### Botox injection leads to decreased expression of pSMAD1/5/8 and VEGF

**Fig 5:**
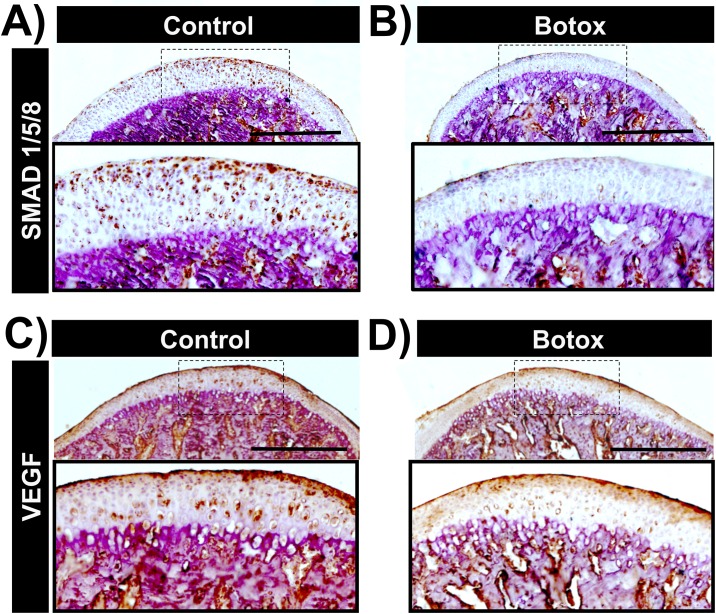
Decreased expression of SMAD1/5/8 and VEFG in the Botox injected side MCC and subchondral bone. Immunohistochemistry for SMAD1/5/8 in sagittal sections of control **(A)** and Botox **(B)** injected side condyles. VEGF Immunohistochemistry in sections of control **(C)** and Botox **(D)** injected side condyles. Scale bar = 500μm.

In order to further understand the mechanism behind the matrix and cellular changes in the MCC and subchondral bone after unilateral injection of Botox into the masseter muscle, we performed immunohistochemistry for pSMAD 1/5/8 and VEGF. Our research has shown increased expression of pSMAD1/5/8 with increased loading of the MCC. BMP2 through pSMAD1/5/8 signaling has been shown to be involved in increased proliferation and hypertrophic differentiation of chondrocytes. Since, we observed a decrease in the number of hypertrophic chondrocytes (by a reduction in Col10a1 expression), a decrease in cell proliferation and increase in apoptosis, we performed immunohistochemistry for pSMAD 1/5/8. We found decreased expression of pSMAD 1/5/8 (Fig 5A and 5B) on the Botox injected side in the prehypertrophic and hypertrophic layers of the MCC and subchondral bone. Furthermore, to understand the decreased bone turnover at the Botox injected side, we performed immunohistochemistry for VEGF, an angiogenic stimulator known for attracting osteoblasts and osteoclasts/chondroclasts during endochondral ossification [19]. There was a decreased expression of VEGF (Fig 5C and 5D) on the Botox injected side in the prehypertrophic and hypertrophic layer of MCC, consistent with reduction in TRAP activity

## Discussion

Botox, a temporary acetylcholine release blocker, has recently been used to alleviate the symptoms of TMD [4,5]. The effects of Botox injections into the masticatory muscles have been reported in animal and clinical investigations [9–12,20,21]. However, studies have not focused on the specific cellular and matrix effects and molecular mechanism behind the detrimental effects of Botox on the subchondral bone and MCC. In this study, we presented the possible cellular and molecular mechanisms associated with mandibular subchondral bone reduction after Botox injection into the masseter muscle. Our objectives were achieved by determining the spatial and temporal changes in Col10a1-RFPcherry expression, tissue remodeling activity (TRAP), mineralization activity of the subchondral bone and MCC (alkaline phosphatase and calcein) and cartilage glycosaminoglycans (Safranin O) and proteoglycans (toluidine blue staining) after Botox injection.

The first reports correlating dimensional changes in the mandible after Botox injections were focused on evaluating the morphological adaptations to decreased muscular force. Kim *et al*. [10] observed smaller mandibular dimensions after unilateral Botox injection into the masseter of growing rats; Tsai *et al*. [20] reported mandibular morphological changes leading to a vertical growth pattern, such as reduced ramus height, increased gonial angle and extrusion of posterior teeth after Botox injection into the masseter of rats. Our morphometric measurements did not show significant differences in mandibular size or condylar height after 4 weeks of unilateral masseter Botox injection, but we found decreased width of the condylar cartilage at the injected side. This observation is consistent with experiments that aimed to alter functional loading of the TMJ of mice by incisor trimming [13], suggesting that Botox injection into the masseter muscle may have an unloading effect in the TMJ.

Muscular loading plays an essential role in maintaining bone volume and density [22]. It has been shown that reducing the volume of thigh and calf muscles by Botox injections cause reduced bone volume and mineral content in the limbs [23–25]. Rafferty *et al*. [11] studied the short-term and long-terms effects of a single Botox injection into the masseter of rabbits and found decreased bone volume of the subchondral bone at the injected side, 4 and 12 weeks after injection, suggesting that the bone loss caused by masseter paralysis persists with time. Tsai *et al*. [26], tested the effects of injecting the temporalis muscle in addition to masseter with Botox, and observed reduced bone mineral density not only in the mandible, but also in the skull of rats. A pilot clinical investigation evaluated cone-beam computed tomography of condyles of women presenting oral-facial pain who were treated with Botox injections into the masticatory muscles or not. All women who received Botox injections presented with decreased subchondral bone density in comparison to women who were not exposed to Botox [9]. These reports are consistent with our findings on reduced bone volume and quality in the subchondral bone of the Botox injected side condyle. Furthermore, our study showed significant decrease in trabecular thickness and increase in trabecular spacing on the injected side when compared to the control.

We next studied bone turnover and mineralization of the MCC and subchondral bone region. TRAP activity was primarily detected in the subchondral bone on the Botox injected side as well as in the control group. However, TRAP activity was significantly less in the Botox group when compared to control. The TRAP enzyme is released by multinucleated osteoclast/chondroclast and activated by cathepsin K [27,28]. Since we found decreased TRAP activity at the Botox injected side, we performed immunohistochemistry for VEGF [19]. We detected VEGF activity in the prehypertrophic and hypertrophic zone of the MCC and in the subchondral bone, but the expression was decreased in the Botox side when compared to control. Reduced TRAP and VEGF activity signifies less bone remodeling in the Botox injected side. In addition, we used alkaline phosphatase assay as an enzymatic indicator of mineralization, but did not find a statistically significant difference in the distance mapping between Botox and control side condyle; but numerically the area of mineralization was more in the control group. Furthermore, bone labeling with calcein and alizarin complexone indicated decreased mineralization at the subchondral bone of the Botox injected side.

The literature is scarce in understanding the cellular changes in the MCC after Botox injections into the masseter muscle. The MCC is formed by fibrocartilage and has the unique capacity to adapt to loading changes [13,17,29]. It has distinct cellular zones expressing different types of collagen and proteins and contains a non-mineralized region rich in proteoglycans for resistance to compressive forces [30]. We observed decreased proteoglycan secretion area in the MCC of the Botox injected side condyle, which is probably a response to decreased loading after masseter paralysis.

The MCC has four distinct cellular zones: 1) superficial or articular zone; 2) proliferative zone composed of undifferentiated mesenchymal cells, which responds to loading demands; 3) prehypertrophic zone, composed of mature chondrocytes expressing Col2; and 4) hypertrophic zone, region where the mature hypertrophic chondrocytes die and undergo calcification. Cells at this last zone express Col10 [29,31]. Our EdU proliferation assay showed significantly decreased cell proliferation in the proliferative zone of MCC of injected side, consistent with adaptation to TMJ unloading [13,29]. Matthys *et al*. [21], studied cell proliferation at the MCC of rabbits that received unilateral Botox injection using BrdU assay, but found no difference in proliferation after Botox injection. The discrepancy between our cell proliferation results and Matthys e*t al*. [21] could be due to differences in the age of experimental animals: our sample consisted of 5-week-old mice, which are considered growing animals, while they used 5-month-old rabbits, animals in maturation age. Younger experiment animals could be more sensitive to changes in cell proliferation than older animals. Cell death is a physiological event associated with transition from chondrogenesis to osteogenesis in the MCC [29], however, we found significant increased TUNEL positive cells at the Botox injected side. Similar results were also reported by Kim *et al*. [10].

To study the changes in Col10a1 expression we used transgenic mice expressing Col10a1-RFPcherry. We found significantly decreased numbers of Col10a1 positive at the hypertrophic zone of Botox injected side in comparison to control. Col10 is an important promoter of endochondral mineralization by regulating matrix mineralization [32]. pSMAD 1/5/8 signaling pathway are crucial in regulation of different phases of chondrogenesis, including chondrocyte proliferation, extracellular matrix deposition and terminal differentiation. Loss/under expression of pSMAD 1/5/8 signaling has shown less proliferation and terminal differentiation [33]. Our immunohistochemistry showed less expression of pSMAD 1/5/8 in the MCC on the Botox injected side when compared to the control; this decrease expression of pSMAD could have resulted in decreased proliferation (EdU staining), decreased terminal differentiation (Col10a1 cells) and decreased proteoglycan and glycosaminoglycans distribution.

To our knowledge, this is the first report regarding the cellular effects of unilateral Botox injection into the masseter muscle in the MCC and the mechanisms behind the detrimental effects of this treatment in the subchondral bone. A limitation of this study was the use of growing mice (5-week-old), however, studies depicting the effects of Botox on the MCC in young mice have yet not been published. Our future studies are focusing on the effects of Botox on MCC in adult and aged mice model. Further studies are necessary to clarify the long-term effects of Botox injections into the masticatory muscles in the MCC in, and also the effects of multiple injections and concomitant injections in additional muscles, such as the temporalis muscle. Furthermore, we will focus on injecting Botox on an established murine TMD model to study the effects of Botox in TMDs.

In conclusion, unilateral Botox injection in masseter muscle leads to decreased mineralization and matrix deposition; cellular changes characterized by reduced chondrocyte proliferation and differentiation and increased cell apoptosis in the MCC and subchondral bone.

## Supporting Information

S1 File Histological characterization of saline injected mice. (DOCX)

S2 File Histological analysis and quantification. (DOCX)

S1 Fig Col10a1 expression, TRAP distribution and proteoglycan secretion in condyles of saline injected mice. Sagittal sections of condyles of saline injected mice. Col10a1 expression and calcein labeling **(A)**, TRAP staining **(B)** and Toluidine Blue staining **(C)**.
(TIF)

S2 Fig Histological quantification. **A)** Red pixels selected using Adobe Photoshop for quantification of Col10a1 positive cells in the MCC area. **B)** Yellow pixels selected for quantification of TRAP positive cells in the subchondral bone. **C)** Toluidine blue stained area quantified. **D)** Quantification of toluidine blue distance mapping.
(TIF)
