# Supplementary material for: Cellular and Matrix Response of the Mandibular Condylar Cartilage to Botulinum Toxin
Source: PLoS One. 2016 Oct 10;11(10):e0164599. doi: 10.1371/journal.pone.0164599 (PMC5056741; doi:10.1371/journal.pone.0164599)
Supplement: S1 File — (DOCX) [file pone.0164599.s001.docx]

**Histological characterization of saline injected mice**

We injected 0.9% saline solution (30 µl) into the right masseter of four animals in our first pilot experiment to differentiate between the effects of Botox and saline solution injection into the masseter. We did not observe differences in Col10a1 expression, TRAP distribution and proteoglycan secretion in the MCC and subchondral bone of saline injected and control side of Botox injected mice (S1 Fig).
