# Supplementary material for: Cellular and Matrix Response of the Mandibular Condylar Cartilage to Botulinum Toxin
Source: PLoS One. 2016 Oct 10;11(10):e0164599. doi: 10.1371/journal.pone.0164599 (PMC5056741; doi:10.1371/journal.pone.0164599)
Supplement: S2 File — (DOCX) [file pone.0164599.s002.docx]

**Histological analysis and quantification**

Col10a1 expression in sagittal sections of condyles were quantified using Adobe Photoshop (Adobe Systems Incorporated, San Jose, CA) by counting the red pixels within the MCC (S2 Fig.). A percentage of red pixels in the MCC were obtained by dividing the number of red pixels over the total number of pixels of entire MCC. We examined TRAP activity in the MCC and subchondral bone by counting the number of yellow pixels (generated by ELF97, S2 Fig.) and dividing it by the total number of pixels in the subchondral bone region. Cell proliferation was quantified by counting EdU and DAPI positive pixels in the proliferative zone of the MCC and then calculating the percentage of EdU positive pixels over DAPI positive pixels. Similarly, cell apoptosis was quantified by calculating the percentage of TUNEL positive pixels over DAPI positive pixels in the MCC.

Alkaline Phosphatase (AP) staining was performed using a fluorescent fast red substrate (SigmaFast^TM^, Sigma, St. Louis, MO) and DAPI. Distance mapping was analyzed using Digimizer^®^ Image software and measurements were performed from the outer cellular layer of MCC to the tidemark (in six different locations in the entire MCC).

Finally, sections were stained with Toluidine Blue (TB) to evaluate proteoglycan secretion within the MCC. TB stained area stained and distance mapping (S2 Fig.) were also evaluated using Digimizer^®^ Image software.
