# Supplementary figures and images for: Cellular and Matrix Response of the Mandibular Condylar Cartilage to Botulinum Toxin
Source: PLoS One. 2016 Oct 10;11(10):e0164599. doi: 10.1371/journal.pone.0164599 (PMC5056741; doi:10.1371/journal.pone.0164599)

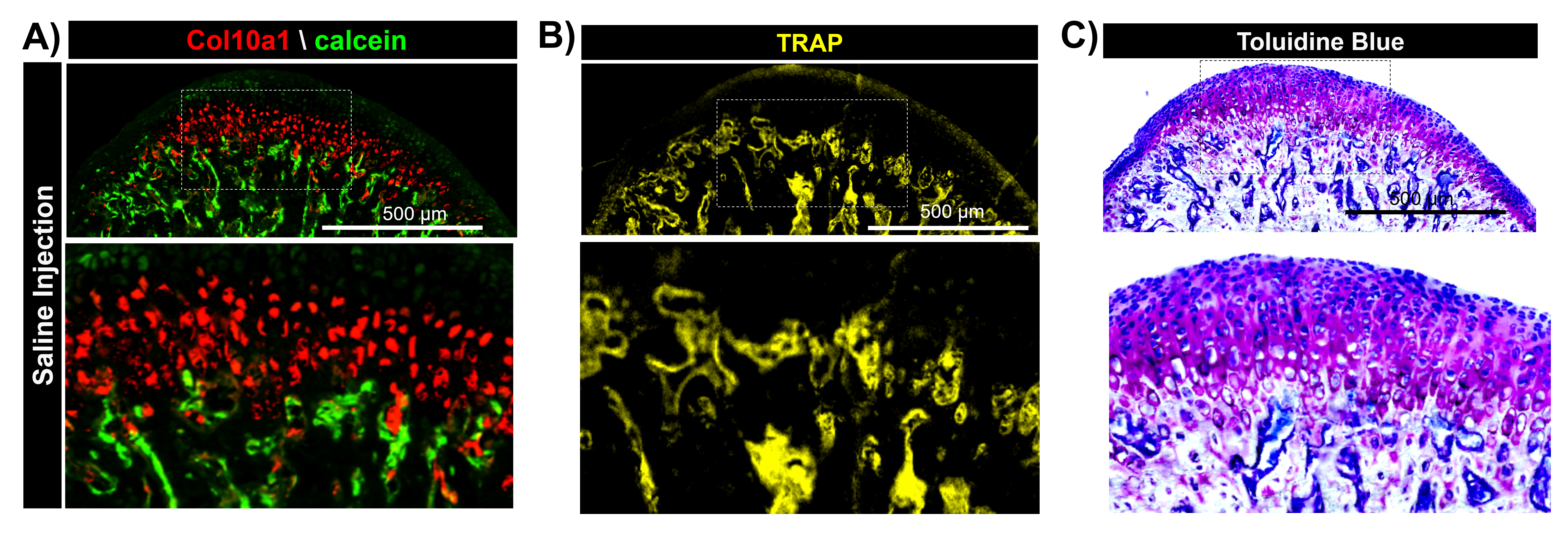

Supplement: S1 Fig — Sagittal sections of condyles of saline injected mice. Col10a1 expression and calcein labeling (A), TRAP staining (B) and Toluidine Blue staining (C). (TIF) [file pone.0164599.s003.tif]

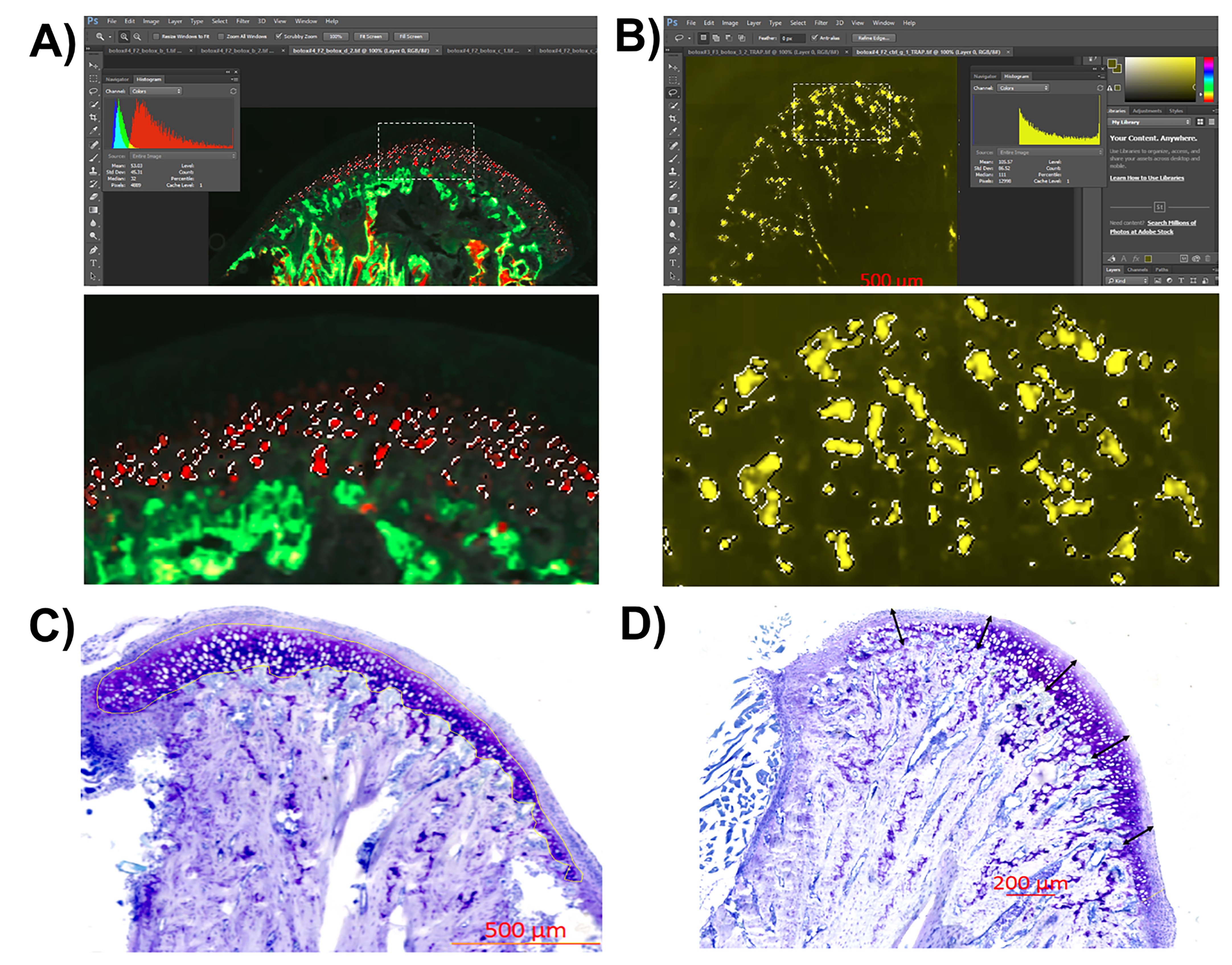

Supplement: S2 Fig — A) Red pixels selected using Adobe Photoshop for quantification of Col10a1 positive cells in the MCC area. B) Yellow pixels selected for quantification of TRAP positive cells in the subchondral bone. C) Toluidine blue stained area quantified. D) Quantification of toluidine blue distance mapping. (TIF) [file pone.0164599.s004.tif]
